# Supplementary material for: Culicoides jiangchengensis, a new species of the subgenus Sinocoides (Diptera, Ceratopogonidae) based on integrative taxonomy from China
Source: PLoS One. 2023 Jul 26;18(7):e0287266. doi: 10.1371/journal.pone.0287266 (PMC10370730; doi:10.1371/journal.pone.0287266)
Supplement: S1 File — (DOCX) [file pone.0287266.s001.docx]

The English in this document has been checked by at least two professional editors, both native speakers of English. For a certificate, please see:

<http://www.textcheck.com/certificate/WoMsXU>
