# Supplementary material for: Culicoides jiangchengensis, a new species of the subgenus Sinocoides (Diptera, Ceratopogonidae) based on integrative taxonomy from China
Source: PLoS One. 2023 Jul 26;18(7):e0287266. doi: 10.1371/journal.pone.0287266 (PMC10370730; doi:10.1371/journal.pone.0287266)

# PLOS ONE

## Culicoides jiangchengensis, a new species of the subgenus Sinocoides (Diptera, Ceratopogonidae) based on integrative taxonomy from China

--Manuscript Draft--

| | |
|---|---|
| **Manuscript Number:** | PONE-D-22-32078 |
| **Article Type:** | Research Article |
| **Full Title:** | Culicoides jiangchengensis, a new species of the subgenus Sinocoides (Diptera, Ceratopogonidae) based on integrative taxonomy from China |
| **Short Title:** | Culicoides jiangchengensis, a new species of the subgenus Sinocoides |
| **Corresponding Author:** | Jinglin Wang, M.D.<br>Yunnan Animal Science and Veterinary Institute<br>Kunming , CHINA |
| **Keywords:** | subgenus Sinocoides, a new species, morphological and molecular identification |
| **Abstract:** | Culicoides have important medical and veterinary significance because their blood-sucking feeding habits enable them to transmit a variety of disease-causing pathogens. In this study, The morphological characteristics of the new species of Culicoides (Sinocoides) jiangchengensis Wang et Liu sp. nov. both sexes are described and compared in detail with the other species of female Culicoides in the subgenus Sinocoides. Three morphological characteristics of C. jiangchengensis, such as without sensory pit in 3rd palpus segment, sensilla coeloconica on flagellomeres 1,9-13, and M1 and M2 cell of the wings with pale spots, were different from the other nine species of culicoides in subgenus Sinocoides. Genetically, C. jiangchengensis most closely related to C. malipoensis, but they were located in different branches and the minimum interspecific distance between them was 12.6%. In addition, a checklist of 10 species in the subgenus Sinocoides Chu, 1983 (Diptera: Ceratopogonidae: Culicoides ) in China, including the new species C. jiangchengensis Wang et Liu sp. nov., is provided, and an updated key to species of the subgenus Sinocoides Chu, 1983 was presented. |
| **Order of Authors:** | Jinglin Wang, M.D. |
| | Yuwen He |
| | Jinxin Meng |
| | Nan Li |
| | Zhao Li |
| | Taoying Yu |
| | Laxi Zhang |
| | Dongmei Wang |
| | Guoping Liu |
| **Additional Information:** | |
| **Question** | **Response** |
| **Financial Disclosure**<br><br>Enter a financial disclosure statement that describes the sources of funding for the work included in this submission. Review the submission guidelines for detailed requirements. View published research articles from *PLOS ONE* for specific examples. | This work is supported by grants from the Basic Research Projects of Yunnan Province (grant no. 2019FA015 and 202201AS070062), National Natural Science Foundation of China (grant no. 32260896 and 81960605), Scientific and technological innovation team construction project of Kunming Medical University (grant no. CXTD202111), Projects funded by the central government to guide local scientific and Technological Development (202207AB110006), and Yunnan Chenggong expert workstation (202005AF150034). The funders had no role in the study design, data collection and analysis, decision to publish, or preparation of the manuscript. |

Powered by Editorial Manager® and ProduXion Manager® from Aries Systems Corporation

| | |
|---|---|
| This statement is required for submission and **will appear in the published article** if the submission is accepted. Please make sure it is accurate.<br><br>**Unfunded studies**<br>Enter: *The author(s) received no specific funding for this work.*<br><br>**Funded studies**<br>Enter a statement with the following details:<br>• Initials of the authors who received each award<br>• Grant numbers awarded to each author<br>• The full name of each funder<br>• URL of each funder website<br>• Did the sponsors or funders play any role in the study design, data collection and analysis, decision to publish, or preparation of the manuscript?<br>• **NO** - Include this sentence at the end of your statement: *The funders had no role in study design, data collection and analysis, decision to publish, or preparation of the manuscript.*<br>• **YES** - Specify the role(s) played.<br><br>* typeset | |
| **Competing Interests**<br><br>Use the instructions below to enter a competing interest statement for this submission. On behalf of all authors, disclose any competing interests that could be perceived to bias this work—acknowledging all financial support and any other relevant financial or non-financial competing interests.<br><br>This statement is required for submission and **will appear in the published article** if the submission is accepted. Please make sure it is accurate and that any funding sources listed in your Funding Information later in the submission form are also declared in your Financial Disclosure statement.<br><br>View published research articles from | The authors have declared that no competing interests exist. |

| | |
|---|---|
| *PLOS ONE* for specific examples.<br><br>**NO authors have competing interests**<br><br>Enter: *The authors have declared that no competing interests exist.*<br><br>**Authors with competing interests**<br><br>Enter competing interest details beginning with this statement:<br><br>*I have read the journal's policy and the authors of this manuscript have the following competing interests: [insert competing interests here]*<br><br>* typeset | |
| **Ethics Statement**<br><br>Enter an ethics statement for this submission. This statement is required if the study involved:<br><br>• Human participants<br>• Human specimens or tissue<br>• Vertebrate animals or cephalopods<br>• Vertebrate embryos or tissues<br>• Field research<br><br>Write "N/A" if the submission does not require an ethics statement.<br><br>General guidance is provided below. Consult the submission guidelines for detailed instructions. **Make sure that all information entered here is included in the Methods section of the manuscript.** | No specifc permits were required for the field studies. After explanation of the purposes and activities of the study, oral consent was obtained from the local participating residents prior to Culicoides collection. No sites were protected by law and this study did not involve endangered or protected species. |

*Powered by Editorial Manager® and ProduXion Manager® from Aries Systems Corporation*

| | |
|---|---|
| **Format for specific study types**<br><br>**Human Subject Research (involving human participants and/or tissue)**<br>• Give the name of the institutional review board or ethics committee that approved the study<br>• Include the approval number and/or a statement indicating approval of this research<br>• Indicate the form of consent obtained (written/oral) or the reason that consent was not obtained (e.g. the data were analyzed anonymously)<br><br>**Animal Research (involving vertebrate animals, embryos or tissues)**<br>• Provide the name of the Institutional Animal Care and Use Committee (IACUC) or other relevant ethics board that reviewed the study protocol, and indicate whether they approved this research or granted a formal waiver of ethical approval<br>• Include an approval number if one was obtained<br>• If the study involved *non-human primates*, add *additional details* about animal welfare and steps taken to ameliorate suffering<br>• If anesthesia, euthanasia, or any kind of animal sacrifice is part of the study, include briefly which substances and/or methods were applied<br><br>**Field Research**<br><br>Include the following details if this study involves the collection of plant, animal, or other materials from a natural setting:<br>• Field permit number<br>• Name of the institution or relevant body that granted permission | |
| **Data Availability**<br><br>Authors are required to make all data underlying the findings described fully available, without restriction, and from the time of publication. PLOS allows rare exceptions to address legal and ethical concerns. See the PLOS Data Policy and FAQ for detailed information. | Yes - all data are fully available without restriction |

| | |
|---|---|
| A Data Availability Statement describing where the data can be found is required at submission. Your answers to this question constitute the Data Availability Statement and **will be published in the article**, if accepted.<br><br>**Important:** Stating 'data available on request from the author' is not sufficient. If your data are only available upon request, select 'No' for the first question and explain your exceptional situation in the text box.<br><br>Do the authors confirm that all data underlying the findings described in their manuscript are fully available without restriction? | |
| **Describe where the data may be found in full sentences. If you are copying our sample text, replace any instances of XXX with the appropriate details.**<br><br>• If the data are **held or will be held in a public repository**, include URLs, accession numbers or DOIs. If this information will only be available after acceptance, indicate this by ticking the box below. For example: *All XXX files are available from the XXX database (accession number(s) XXX, XXX.)*.<br>• If the data are all contained **within the manuscript and/or Supporting Information files**, enter the following: *All relevant data are within the manuscript and its Supporting Information files.*<br>• If neither of these applies but you are able to provide **details of access elsewhere**, with or without limitations, please do so. For example:<br><br>*Data cannot be shared publicly because of [XXX]. Data are available from the XXX Institutional Data Access / Ethics Committee (contact via XXX) for researchers who meet the criteria for access to confidential data.*<br><br>*The data underlying the results presented in the study are available from (include the name of the third party* | All sequence are available from the accession number(s) OL471017-OL471027. |

Powered by Editorial Manager® and ProduXion Manager® from Aries Systems Corporation

| | |
|---|---|
| *and contact information or URL).*<br>• This text is appropriate if the data are owned by a third party and authors do not have permission to share the data.<br><br>* typeset | |
| Additional data availability information: | |

Manuscript
Click here to access/download;Manuscript;Culicoides jiangchengensis-20221121.docx

# *Culicoides jiangchengensis*, a new species of the subgenus *Sinocoides* (Diptera, Ceratopogonidae) based on integrative taxonomy from China

Yuwen He[1], Jinxin Meng[1], Nan Li[1], Zhao Li[2], Yu Taoying[3], Laxi Zhang[2], Dongmei Wang [2], Guoping Liu[4], Jinglin Wang[1]

**1** Yunnan Tropical and Subtropical Animal Viral Disease Laboratory, Yunnan Animal Science and Veterinary Institute, Kunming 650224, China **2** Jiangcheng County Animal Disease Prevention and Control Center, Jiangcheng 665900，China **3** Gongshan County Animal Disease Prevention and Control Center, Gongshan 673500, China **4** Center for Disease Control and Prevention of Shenyang Command, Shenyang 110034, China

Corresponding author: Jinglin Wang (Wangjl107@163.com), Guoping Liu (kqhxj2@163.com)

Abstract: 102 words

Manuscript: 2391 words, Tables 2, Figures, 11

*Culicoides* have important medical and veterinary significance because their blood-sucking feeding habits enable them to transmit a variety of disease-causing pathogens. In this study, The morphological characteristics of the new species of *Culicoides* (*Sinocoides*) *jiangchengensis* Wang et Liu sp. nov. both sexes are described and compared in detail with the other species of female Culicoides in the subgenus *Sinocoides*. Three morphological characteristics of *C. jiangchengensis*, such as without sensory pit in 3$^{rd}$ palpus segment, sensilla coeloconica on flagellomeres 1,9-13, and $M_1$ and $M_2$ cell of the wings with pale spots, were different from the other nine species of culicoides in subgenus *Sinocoides.* Genetically, *C. jiangchengensis* most closely related to *C. malipoensis*, but they were located in different branches and

the minimum interspecific distance between them was 12.6%. In addition, a checklist of 10 species in the subgenus *Sinocoides* Chu, 1983 (*Diptera: Ceratopogonidae*: *Culicoides*) in China, including the new species *C. jiangchengensis* Wang et Liu sp. nov., is provided, and an updated key to species of the subgenus *Sinocoides* Chu, 1983 was presented.

**Keywords**: subgenus *Sinocoides*, a new species, morphological and molecular identification

## Introduction

Biting midges are important vector insects that transmit arbovirus diseases such as Oropouche virus, bluetongue virus (BTV), epizootic haemorrhagic disease virus (EHDV), African horse sickness virus (AHSV), Ibaraki disease virus (IBADV), Schmallenberg virus (SBV), Bovine ephemeral fever virus (BEFV), and vesicular stomatitis virus (VSV) by biting humans, livestock, poultry, and other animals (Carpenter et al. 2013; Sakkas et al. 2018; Rozo et al. 2020). Currently, 1764 species of bloodsucking midge are classified in four genera worldwide, of which *Culicoides* Latreile (Didptera, Ceratopogonidae) is the largest, including 1399 (79.31%) species in 38 species groups in 33 subgenera (Borkent and Dominiak 2020). In China, there are 451 species of bloodsucking midge in three genera, and *Culicoides* includes 336 species in 12 subgenera (Yu et al. 2015; Yang et al. 2020). Qu (1983) established the subgenus *Sinocoides* Chu, 1983 in genus *Culicoides*. At that time, only one species of this subgenus was described in China, C*ulicoides hamiensis* Qu, Qian et Ma, 1982 (Qu et al. 1982). Subsequently, eight new species in the subgenus were described by Liu et al. (1990, 1996, 2003) and Yu et al. (2005). In the last decade, Liu et al. (2006, 2011) and Wu et al. (2018) described four new species in this subgenus. Here, a new species of the subgenus *Sinocoides* is described in an investigation on blood-sucking midges in Yunnan Province, China. Cytochrome c oxidase subunit Ⅰ gene (COI) sequences were obtained to identify the species of *Culicoides*. The main distinguishing characteristics, geographical distribution, and a species checklist of the 10 species of this subgenus found in China are reported.

## Materials and methods

Biting midges were collected overnight from 7:00 pm to 7:00 am the next morning in domestic animal pens using light traps (12 V, 300 mA; Wuhan Lucky Star Environmental Protection, Hubei, China) in Jiangcheng County in September 2015 and Gongshan County in august 2017 of Yunnan Province, china. The midges were stored in 70% ethanol at 4℃ and immersed in 250 µL tissue digestive solution with 1% proteinase K (TIANGEN DNA extraction kit) for non-destructive tissue digestion. The midges were slide-mounted in Canada balsam, as described by Yu et al. (2005). Midge genomic DNA was extracted from the digestive supernatant (Yuan et al. 2019), and a partial mitochondrial cytochrome c oxidase I (COI) gene sequence was obtained by PCR amplification and sequencing (Cêtre-Sossah et al. 2004). The COI gene sequence was submitted to GenBank under accession number OL471017-OL471027 and analyzed using MEGA-X software.

## Taxonomy

***Culicoides*** **(*Sinocoides*)** ***jiangchengensis Wang et Liu*** **sp. nov. (Figure 1)**

http://ZooBank.org/References/E8649BF5-471C-4812-A61E-360486C59946

**Diagnosis**. Females: only for *Culicoides* species in Yunnan with the following combination of features: palpus third segment slightly swollen at distal 1/3, without sensory pit, with sensilla scattered on the surface; a small, approximately oval, fuzzy pale spot at the distal end of $r_5$ cell; $m_1$ with two pale spots, anal cell with a wide pale band extended from its base to distal; three equally sized spermathecae. Male: only for *Culicoides* species in Yunnan with the following combination of features: palpus third segmentwithout sensory pit; anal cell with a big, long irregular pale spot shaped like a pocket; The arch of aedeagus with deep bow type, the middle of aedeagus reflexed in apex, parameres separate, the apex part bends in a hook shape.

**Description:**

**Female**

**Body** (Figure 1A) Small and medium-sized midges. Wing length 1.08 (1.0–1.22) mm, breadth 0.48 mm, CR 0.50 (0.48–0.53, n = 9).

**Head** (Figure 1C,D,E,F,G) Brown. Eyes bare, separated by distance approximately equal to the diameter of one ommatidium, and a transverse seam in the lower forehead. Antenna pedicel slightly pale brown; flagellomeres 1-8 short and stout, flagellomeres 9-13 longer, lengths of flagellomeres in the proportion of 19:19:19:19:19:19:18:19:26:28:34:35:55; AR 1.13 (1.08–1.18, n = 9); sensilla coeloconica on flagellomere 1, 9–13. Palpus five-segmented, lengths in the proportion of 10:20:22:12:13, segment 3 slightly swollen at apical 1/3, without sensory pit, with sensilla scattered on the surface; PR 2.63 (2.50–2.75, n = 9). Three bristles on each side of the frontoclypeus. Proboscis length 150 μm, head height 250 μm, P/H ratio 0.59 (0.53–0.64, n = 9); mandible with 15–17 teeth (n = 9), maxilla with 14 teeth (n = 9).

**Thorax** (Figure 1J,K) Scutum light brown, scutellum dark brown; wing with contrasting pattern of pale/dark spots; wing base with a large pale spot and connected to the wide pale band of the anal cell; basal 2/3 of 1$^{st}$ radial cell and r-m cross-vein in pale spot, and extension posterior over M1 to connect with narrow pale band of $m_2$; distal 4/5 of 2$^{nd}$ radial cell covered by pale spot; apical pale spot in cell $r_5$ cell faint, and reaching wing margin; $m_1$ with two pale spots, proximal pale spot long band; $m_2$ with two differently shaped pale spots: proximal pale spot long band extending from the base to distal, distal pale spot abutting the wing margin; $m_4$ with a pale spot abutting wing margin; anal cell with a wide pale band extending from base to distal; macrotrichia on the costal of the proximal of $R_5$ cell and distal of $m_1$, but no on the proximal of $m_1$; hind tibial comb with four spines, 2$^{nd}$ spine longest, metatibial comb (mc) about 18 teeth, TR and F-T of legs are as in Table 1.

**Abdomen** Light brown. Three subequal-size ovoid spermathecae (Figure 1L), each measuring 25.00×23.00 μm.

**Male** (Figure 1B) Similar to female with usual sexual differences.

**Head** The lengths of Antenna flagellomeres in the proportion 26:13:13:14:14:14:12:11:10:9:34:33:38 , sensilla coeloconica on flagellomeres 1, 11-13; palpus with segments in proportions of 8:10:11:7:9, PR 1.57 (n=1); one bristles on each side of the frontoclypeus.

**Thorax** Wing with pattern of pale spots as in Fig. 1b, wing length 0.82 (n = 1), width 0.33 (n=1); CR 0.63 (n=1); wings light dark, with eight pale spot; wing costal with three obvious dark spots; basal 1/3 of 1st radial cell and r-m cross-vein in pale spot; distal 2/3 of 2nd radial cell covered by pale spot; anal cell with a big irregular pale spot. Metatibial comb (mc) about 18 teeth, TR and F-T of legs are given as Table 1.

**Genitalia** (Figure 1M,N): the middle of the posterior margin of the 9$^{th}$ genitalia sternite concave, wide, arc and the membrane free of microhairs. The posterior margin 9th basal tergite flat, a small V-shaped concave was observed in the middle part, parameres robust, sharp Angle shape. Gonocoxite slender in its basal part, the dorsal ankle digitation, mucro part bend to the medial side. The middle aedeagus is nearly tapered, aedeagus with deep bow type, the arch height of the aedeagus is about 1/2 of the total length of the aedeagus, reflexed in apex. Parameres separated, the middle part thick, diminution, curve and with a hook shape in apex.

**Etymology.** The name jiangchengensis refers to the collecting location of the species.

**Type material.** Holotype female, Qiaotouhe Village, Menglie Town, Jiangcheng County, Yunnan Province, China (22°54′49″N, 101°88′70″E), September 2015, light trap. Male, Maxidang Village, Bangdang Town, Gongshan County, Yunnan Province, China (27°49′54.8328″N, 98°41′46.0536″E), Augest 2017, light trap.

**Paratypes:** 9 females and 1 male, same data as holotype.

**Distribution.** China (Jiangcheng County and Gongshan county of Yunnan Province).

**Remarks.** *C. jiangchengensis* collected in Jiangcheng County in this study have three spermathecae, which were similar to those of the subgenus *pontoculicoides*,

*Sinocoides, Jilinocoides* and *Trithecides*. Two eyes of *C. jiangchengensis* were separated, excluding the subgenus *Jilinocoides* and *Trithecides*, and the wings have pale spots and dark spots excluding *pontoculicoides*, indicating that *C. jiangchengensis* is a member of the subgenus *Sinocoides*. At present, there are 9 species of *Culicoides* in subgenus *Sinocoides*, among which *C. anthropophygas* (Figure 2), *C. hamiensis* (Figure 3), *C. jinghongensis* (Figure 4), *C. kongmiaoensis* (Figure 5), *C. multifarious* (Figure 6), *C. opertus* (Figure 7) had an obvious sensory pit in the 3$^{rd}$ segment of palpus, while *C.jiangchengensis* had no an obvious sensory pit, but with capitate sensilla scattered on the surface, which had significant differences between them. Although *C. pungobovis* (Figure 8) and *C. jiangchengensis* have similar morphological characteristics in the 3$^{rd}$ segment of palpus, their sensilla coeloconica on flagellomere are significantly different, the former is 1,6-8, while the latter is 1,9-13. *C. malipoensis* (Figure 9), *C. nanniwanensis* (Figure 10) and *C.jiangchengensis* had similar morphological characteristics in the 3$^{rd}$ palpus segment and the sensilla coeloconica on flagellomere, but there was no pale spot in the wings $m_1$ and $m_4$ of *C. nanniwanensis,* while the $m_1$ and $m_4$ of *C. malipoensis* and *C. jiangchengensis* with pale spot. *C. jiangchengensis* is the most similar to *C. malipoensis* in morphological characteristics, but the former has 2 pale spots in the $m_1$ and a wide pale band extended from the basal to the distal in the anal cell, while the latter has a pale band extended from base to the distal in the $m_1$ and a pale spot at the proximal in the anal cell.

In addition, the main characteristics distinguishing *Culicoides* in China are wing length, costal ratio (CR), antennal ratio (AR), proboscis ratio (PR), mandible teeth, and sensilla coeloconica on flagellomere. Table 1 provides detailed measurements.

**DNA analyses**

The phylogenetic tree based on the COI gene sequences of *C. jiangchengensis*, *C. malipoensis* from Jiangcheng County and Gongshan County, Yunnan Province and another 24 *Culicoides* shows that nine female *C. jiangchengensis* from Jiangcheng

and two male *C. jiangchengensis* from Gongshan formed a separate branch （Figure 11）. The mean intraspecific distance was 1%, and the maximum was 2.36%. Although *C. jiangchengensis* and *C. malipoensis* are on different branches, their genetic relationship is closest among other *Culicoides*; the minimum interspecific distance was 12.6%.

**Checklist of *Sinocoides* in China and their geographical distribution**

There are 10 species in the subgenus *Sinocoides* in China, distributed in eight provinces and autonomous regions of China.

(1) *Culicoides* (*Sinocoides*) *jiangchengensis* Wang et Liu sp. nov. (Figure 1) Type locality: China: Yunnan (Jiangcheng).
Distribution: Yunnan (Jiangcheng, Gongshan).

(2) *Culicoides* (*Sinocoides*) *anthropophygas* Yu et Liu, 2005 (Figure 2)
*Culicoides* (*Sinocoides*) *anthropophygas* Yu et Liu, 2005:892; Type locality: China: Sichuan (Jiulong).
Distribution: Sichuan (Jiulong).

(3) *Culicoides* (*Sinocoides*) *hamiensis* Chu, Qian et Ma, 1982 (Figure 3)
*Culicoides* (*Sinocoides*) *hamiensis* Chu, Qian et Ma, 1982:105; Type locality: China: Xinjiang (Hami).
Distribution: Xinjiang (Hami).

(4) *Culicoides* (*Sinocoides*) *jinghongensis* Wu et Liu, 2018 (Figure 4)
*Culicoides* (*Sinocoides*) *jinghongensis* Wu et Liu, 2018:290; Type locality: China: Yunnan (Jinghong).
Distribution: Yunnan (Jinghong).

(5) *Culicoides* (*Sinocoides*) *kongmiaoensis* Liu et Zhou, 2006 (Figure 5)
*Culicoides* (*Sinocoides*) *kongmiaoensis* Liu et Zhou, 2006: 467; Type locality: China: Shandong (Qiuhu).
Distribution: Shandong (Qiuhu).

(6) *Culicoides* (*Sinocoides*) *multifarious* Liu, Gong et Zhang, 2003 (Figure 6)
*Culicoides* (*Sinocoides*) *multifarious* Liu, Gong et Zhang, 2003:359; Type locality: China: Gansu (Tianshui).
Distribution: Gansu (Tianshui).

(7) *Culicoides* (*Sinocoides*) *opertus* Liu et Yu, 1990 (Figure 7)

*Culicoides* (*Sinocoides*) *opertus* Liu et Yu, 1990:15; Type locality: China: Heilongjiang (Hulin).

Distribution: Heilongjiang (Hulin, Raohe, Suifenhe).

(8) *Culicoides* (*Sinocoides*) *pungobovis* Liu, Yan et Liu, 1996 (Figure 8)

*Culicoides* (*Sinocoides*) *pungobovis* Liu, Yan et Liu, 1996:35; Type locality: China: Hainan (Qiongzhong).

Distribution: Hainan (Qiongzhong).

(9) *Culicoides* (*Sinocoides*) *malipoensis* Liu et Ren, 2011 (Figure 9)

*Culicoides* (*Sinocoides*) *malipoensis* Liu et Ren, 2011:257; Type locality: China: Yunnan (Malipo).

Distribution: Yunnan (Malipo)

(10) *Culicoides* (*Sinocoides*) *nanniwanensis* Liu et Wang, 2011 (Figure 10)

*Culicoides* (*Sinocoides*) *nanniwanensis* Liu et Wang, 2011:257; Type locality: China: Shanxi (Nanniwan).

Distribution: Shanxi (Nanniwan).

**Discussion**

The three main morphological characteristics that identified *C. jiangchengensis* as subgenus *Sinocoides* were the separation of two eyes, capitate sensilla scattered on the surface of 3rd segment palpus, the contrasting pattern of pale/dark spots in the wing, and three subequal-size ovoid spermathecae, which is similar to that of other culicoides of subgenus *Sinocoides*.

According to the following three morphological characteristics of *C. jiangchengensis*: 1 ) without sensory pit in 3rdsegment of palpus; 2 ) sensilla coeloconica on flagellomere 1, 9-13; 3 ) $m_1$ and $m_2$ cell of the wings with pale spots, which were different from the other eight species of culicoides in *subgenus Sinocoides*, such as *C. anthropophygas, C. hamiensis, C. jinghongensis, C. kongmiaoensis, C. miaoensis, C. opertus, C. pungobovis* and *C. nanniwanensis*. *C. jiangchengensis* is morphologically similar to *C. malipoensis*, but *C. malipoensis* is a

medium-sized *Culicoides*, with a wing length of 1.35 mm, mandible with nine teeth, P/H ratio 0.64, the distal of $r_3$ with a large fuzzy pale spot, $m_1$ cell with a wide pale band extending from the base to distal, and distal of anal cell with a pale spot, which is obviously different from *C. jiangchengensis*.

Molecular biology is one of the methods for rapid and accurate identification of vector insect species (Wang 2011). COI gene with moderate evolutionary rate is the most commonly used molecular target for mosquito and midges identification (Wang 2011; Monaghan et al. 2005). In this study, the COI gene was used as a molecular target to identify *C. jiangchengensis* and *C. maripoensis* collected in Jiangcheng and Gongshan. The results showed that *C.jiangchengensis* was mostly related to, but distinct from, *C.malipoensis* in subgenus *Sinocoides*. The minimum interspecific distance between them was 12.6%, higher than between *C. selandicus* and *C. kalix* (5.9%) (Nielsen et al. 2015) and *C. fagineus* F1 and *C. subfagineus* (*sensu stricto*) (12%) within the subgenus *Culicoides* (Pagès et al. 2009) and between *C. bolitinos* and *C. tutti-frutti* (9.5%) within the subgenus *Avaritia* (Augot et al. 2016). These data indicated that *C. jiangchengensis* is a new species in subgenus *Sinocoides* based on its morphology and molecular biology, increasing the number of *Culicoides* species in China. Meanwhile, this is the first time to obtain the sequence of *Culicoides* in subgenus *Sinocoides*, which provides sequence information for rapid molecular identification and genetic evolution of *Culicoides* species in China.

Since subgenus *Sinocoides* was established in China in 1983, females of nine *Culicoides* species in this subgenus have been recorded in different regions of China. This survey discovered a new *Culicoides* species in *Sinocoides* in Yunnan, increasing the species in this subgenus to 10. Based on the literature, a key to the females of 10 *Culicoides* in subgenus *Sinocoides* was compiled. Fortunately, in this study, we used molecular biology methods to identify the males of *C.jiangchengensis*, and systematically described the morphological characteristics of the males, providing data for the classification and identification of *C.jiangchengensis* in the future. However, due to the lack of morphological description of other subgenus Sinocoides

male, failed to compile a key to the males of 10 *Culicoides* in this subgenus, and needs further investigation.

**An updated key to females in the *subgenus Sinocoides* of *Culicoides***

1 Eyes hairy, Wing without pale and dark spot (Figure 2)...... *C.* (*S.*) *anthropophygas*

Eyes bare, Wing with pale and dark spot............................................................ 2

2 (1) $m_1$,$m_2$,$m_4$ cell with pale........................................................................... 3

$m_1$,$m_2$,$m_4$ cell without pale....................................................................... 7

3 (2) $r_5$ cell with 2~3 pale spots except the distal pale................................................ 4

$r_5$ cell with 1 pale spot except the distal pale...................................................... 5

4 (3) $r_5$ cell with 3 pale spots except the distal pale, $m_1$ cell with 2 pale spots (Figure 6) …………………………………………………………..*C.* (*S.*) *multifarious*

$r_5$ cell with 2 pale spots except the distal pale, $m_1$ cell with 3 pale spots (Figure 8).....……………………………………………………… *C.* (*S.*) *pungobovis*

5 (3) $m_2$ cell with a pale band and a pale spot............................................................ 6

$m_2$ cell with 4 pale spots (Figure 4)..........................................*C.* (*S.*) *jinghongensis*

6 (5) $m_1$ cell with pale band extended from base to the distal, anal cell with a pale spot at the Proximal (Figure 9)....................................................*C.* (*S.*) *malipoensis*

m1 cell with 2 pale spots, anal cell with a wide pale band extended from the basal to the distal (Figure 1)...................................................*C.* (*S.*) *jiangchengensis*

7(2) sensilla coeloconica on flagellomeres 1~12 or 1~13............................................. 8

sensilla coeloconica on flagellomeres 1,9~12 or 1,9~13....................................... 9

8 (7) sensilla coeloconica on flagellomeres 1~12; mandible 18 teeth (Figure 3) ...................................................................................................... *C.* (*S.*) *hamiensis*

sensilla coeloconica on flagellomeres 1~13; mandible 13 teeth(Figure 5)...........................................................................................*C.* (*S.*) *kongmiaoensis*

9 (7) sensilla coeloconica on flagellomeres 1,9~12; $m_2$ and anal cell without pale spot (Figure 7) ......................................................................................... *C.* (*S.*) *opertus*

sensilla coeloconica on flagellomeres 1,9~13; $m_2$ and anal cell with a pale spot respectively (Figure 10)............................................................*C.* (*S.*) *nanniwanensis*

**Authors' contributions**

WJL and LGP conceived and designed the experiments. MJX, HYW, LN, and LGP performed the experiments. WJL, MJX, HYW, LZ, YTY, ZLX, WDM collected

Culicoides samples. WJL and LGP analyzed the data. WJL and LGP wrote and revised the manuscript. All authors read and approved the final manuscript

**Acknowledgments**

This work is supported by grants from the Basic Research Projects of Yunnan Province (grant no. 2019FA015 and 202201AS070062), National Natural Science Foundation of China (grant no. 32260896 and 81960605), Scientific and technological innovation team construction project of Kunming Medical University (grant no. CXTD202111), Projects funded by the central government to guide local scientific and Technological Development (202207AB110006), and Yunnan Chenggong expert workstation (202005AF150034). The funders had no role in the study design, data collection and analysis, decision to publish, or preparation of the manuscript.

**Availability of data and materials**

The data sets supporting the results are included within the article. Nucleotide sequence data reported in this paper have been deposited to the NCBI GenBank database under accession numbers OL471017–OL471027.

**Declarations**

**Ethics approval and consent to participate**

No specifc permits were required for the field studies. After explanation of the purposes and activities of the study, oral consent was obtained from the local participating residents prior to *Culicoides* collection. No sites were protected by law and this study did not involve endangered or protected species.

**Consent for publication**

Not applicable.

**Declaration of Competing Interests**

The funders had no role in study design, data collection and analysis, decision to publish, or preparation of the manuscript.

**Author details**

[1] Yunnan Tropical and Subtropical Animal Viral Disease Laboratory, Yunnan Animal Science and Veterinary Institute, Kunming 650224, China. [2] Jiangcheng County Animal Disease Prevention and Control Center, Jiangcheng 665900, China. [3] Gongshan County Animal Disease Prevention and Control Center, Gongshan 673500, China. [4] Center for Disease Control and Prevention of Shenyang Command, Shenyang 110034, China.

## References

Augot D, Hadj-Henni L, Millot C, Lehrter V, Cousinat M, Depaquit J. (2016) First report of *Culicoides boyi* (Diptera: Ceratopogonidae) in France. Ann Société Entomol Fr NS 0:1–8.

Borkent A, Dominiak P. (2020) Catalog of the biting midges of the world (Diptera: Ceratopogonidae) (Zootaxa). Auckland New Zealand: Magnol Pre. 4787 (1):1–377.

Carpenter S, Groschup MH, Garros C, Felippe-Bauer ML, & Purse BV (2013) Culicoides biting midges, arboviruses and public health in Europe. Antiviral research 100(1):102–113.

Cêtre-Sossah C, Baldet T, Delécolle JC, Mathieu B, Perrin A, Grillet C, Albina E. (2004) Molecular detection of *Culicoides* spp. and *Culicoides imicola*, the principal vector of bluetongue (BT) and African horse sickness (AHS) in Africa and Europe. Vet Res. 35(3):325–37. doi: 10.1051/vetres:2004015.

Liu GP, Yu YX. (1990) Three new species of blood-sucking in Northeastern China. Contr blood-sucking Dipt. Ins. Shanghai: Shanghai Science Press (2):14–18.

Liu JH, Yan G, Liu GP. (1996) The biting midge from Hainan Island. Beijing: Milit Med Sci Pre 14–184.

Liu ZJ, Gong ZW, Zhang JJ, Shi SZ. (2003) Two new species of Culicoides (Diptera: Ceratopogonidae). Acta Zootaxonomica Sinica 28(2):359–361.

Liu GP, Zhou X. (2006) *Culicoides* (*Sinocoides*) and A new species from China (Diptera: Ceratopogonidae). Chin J Vector Biol & Control 17(6):467–469.

Liu GP, Ren QM, Wang X. (2011) Culicoides (Sinocoides) and two new species from China (Diptera: Ceratopogonidae). Chin J Vector Biol & Control 22(3):257–259.

Monaghan M., Balke M, Gregory TR, et al. (2005).DNA-based species delineation in tropical beetles using mitochondrial and nuclear markers. Phil. Trams. R. Soc.B, 360: 1925-1933.

Nielsen SA, Kristensen M.(2015) Delineation of Culicoides species by morphology and barcode exemplified by three new species of the subgenus Culicoides (Diptera: Ceratopogonidae) from Scandinavia. Parasit Vectors 8:151.

Pagès N, Munoz-Munoz F, Talavera S, Sarto V, Lorca C, Nunez JI. (2009) Identification of cryptic species of Culicoides (Diptera: Ceratopogonidae) in the subgenus Culicoides and development of species-specific PCR assays based on barcode regions. Vet Parasitol 165:298–310.

Qu FY.(1983) Two new subgenera and two new species of Culicoides from China (Diptera: Ceratopogonidae). Entomotaxonoia 5(1):25–32.

Qu FY, Qian JQ, Ma DX.(1982) Records of biting midges collected from Xinjiang, China, with descriptions of two new species (Diptera: Ceratopogonidae). Acta Entomology Sinica 25(1):105–110.

Rozo-Lopez P, Londono-Renteria B, Drolet BS (2020) Venereal transmission of vesicular stomatitis virus by *Culicoides sonorensis* midges. Pathogens 9(4):316.

Sakkas H, Bozidis P, Franks A, Papadopoulou C (2018) Oropouche fever: A review. Viruses 10(4):175.

Wang G. (2011) Molecular identification and Phylogeny of China main mosquito species based on DNA Barcoding. Academy of Military Sciences. Doctoral Dissertation.

Wu CG, Liu GP. (2018) Classification of Culicoides (Sinocoides) and a new species from China (Diptera: Ceratopogonidae). Chin J Vector Biol & Control 29(3):290–292 . DOI:10.11853/j.ssn.1003.8280.2018.03.019.

Yu YX, Liu JH, Liu GP. (2005) Ceratopogonida of China. Vol. Ⅰ Ⅱ. Beijing: Milit Med Sci Pre 43–114,692–802,816–1322.

Yuan ZJ, Bai F, Li Zhao, He YW, Meng JX, Li N, Zhang NX, Wang JL. (2019) Morphological and molecular biological identification of a new species of *Allohelea* from Yunnan border area. Chinese Frontier Health Quarantine 42(1):29–32.

Yang D, Li Z, Liu QF. (2020) Species catalogue of China, Vol 2 Animals, Insecta (V) . Beijing: Science Press, 119–221.

**Figure Legends**

**Figure 1** *Culicoides* (*Sinocoides*) *jiangchengensis* Wang et Liu, sp. nov. A: Female; B:Male; C:Head; D:front; E: antenna; F:maxillary palpus; G:mandibles; H:wing (Female); I:wing(Male); J:Thorax; K:hind tibial comb; L:spermatheca; M:hypopygium; N: parameres.

**Figure 2** *Culicoides* (*Sinocoides*) *anthropophygas* Yu et Liu, 2005. ant, antenna; f, front; map, maxillary palpus; md, mandibles; spt, spermatheca; w, wing.

**Figure 3** *Culicoides* (*Sinocoides*) *hamiensis* Chu, Qian et Ma, 1982. ant, antenna; f, front; map, maxillary palpus; md, mandibles; spt, spermatheca; w, wing.

**Figure 4** *Culicoides* (*Sinocoides*) *jinghongensis* Wu et Liu, 2018. ant, antenna; f, front; map, maxillary palpus; md, mandibles; spt, spermatheca; w, wing.

**Figure 5** *Culicoides* (*Sinocoides*) *kongmiaoensis* Liu et Zhou, 2006. Ant, antenna; f, front; map, maxillary palpus; md, mandibles; spt, spermatheca; w, wing.

**Figure 6** *multifarious* Liu, Gong et Zhang, 2003. ant, antenna; f, front; map, maxillary palpus; md, mandibles; spt, spermatheca; w, wing.

**Figure 7** *Culicoides* (*Sinocoides*) *opertus* Liu et Yu, 1990. ant, antenna; f, front; map, maxillary palpus; md, mandibles; spt, spermatheca; w, wing.

**Figure 8** *Culicoides* (*Sinocoides*) *pungobovis* Liu, Yan et Liu , 1996. ant, antenna; f, front; map, maxillary palpus; md, mandibles; spt, spermatheca; w, wing.

**Figure 9** *Culicoides* (*Sinocoides*) *malipoensis* Liu et Ren, 2011. ant, antenna; f, front; map, maxillary palpus; md, mandibles; spt, spermatheca; w, wing.

**Figure 10** *Culicoides* (*Sinocoides*) *nanniwanensis* Liu et Wang, 2011. ant, antenna; f, front; map, maxillary palpus; md, mandibles; spt, spermatheca; w, wing.

**Figure 11** ML phylogenetic trees of COI gene nucleotide sequences of *C.* (*Avaritia*) *jiangchengensis* from Jiangcheng County and Gongshan County, Yunnan Province, China using MAGE-X. The best DNA substitution model is GTR+G+I, and the trees were drawn using njplot 2.4 with the bootstrap value based on 1,000 replications.

**Table 1** Tarsal ratios (TR) and measurements of leg segments and tarsomeres from femur to tarsomere 5 (F-T) of all legs of *Culicoides* (*S*) *jiangchengensis* Wang et Liu sp. nov.

| | Leg | TR | F-T |
|---|---|---|---|
| Female(♀) | Foreleg | 2.71 | 78:78:38:14:10:8:10 |
| | Midleg | 2.89 | 97:101:52:18:12:13:9 |
| | Hindleg | 1.68 | 92:95:49:23:15:9:10 |
| Male(♂) | Foreleg | 2.42 | 58:51:29:12:9:8:9 |
| | Midleg | 3.17 | 72:67:38:12:10:7:8 |
| | Hindleg | 2.07 | 66:65:31:15:10:8:9 |

**Table 2** Measurements of distinguishing characteristics of *Culicoides* (*Sinocoides*) in China (Female)

| species | wing length (mm) | CR | AR | PR | mandible teeth | antennal sensilla pattern |
|---|---|---|---|---|---|---|
| *C. anthropophygas* | 1.35 | 0.61 | 1.29 | 2.50 | 14 | 3, 9~15 |
| *C. hamiensis* | 1.18 | 0.57 | 0.96 | 2.50 | 18 | 3~14 |
| *C. jiangchengensis* | 1.08 | 0.50 | 1.13 | 2.63 | 17 | 3, 11~15 |
| *C. jinghongensis* | 1.08 | 0.54 | 0.96 | 1.70 | 12 | 3, 7~10 |
| *C. kongmiaoensis* | 1.18 | 0.53 | 1.59 | 2.42 | 13 | 3~15 |
| *C. multifarious* | 0.97 | 0.55 | 0.98 | 2.44 | 13 | 3, 8~10 |
| *C. malipoensis* | 1.35 | 0.70 | 1.05 | 2.90 | 9 | 3, 11~15 |
| *C. nanniwanensis* | 1.05 | 0.57 | 1.18 | 2.33 | 15 | 3, 11~15 |
| *C. opertus* | 1.13 | 0.59 | 1.20 | 2.50 | 18 | 3, 11~14 |
| *C. pungobovis* | 0.78 | 0.58 | 0.82 | 2.00 | 17 | 3, 8~10 |

The English in this document has been checked by at least two professional editors, both native speakers of English. For a certificate, please see:

http://www.textcheck.com/certificate/WoMsXU

Figure 1

Click here to access/download;Figure;Figure 1.tif

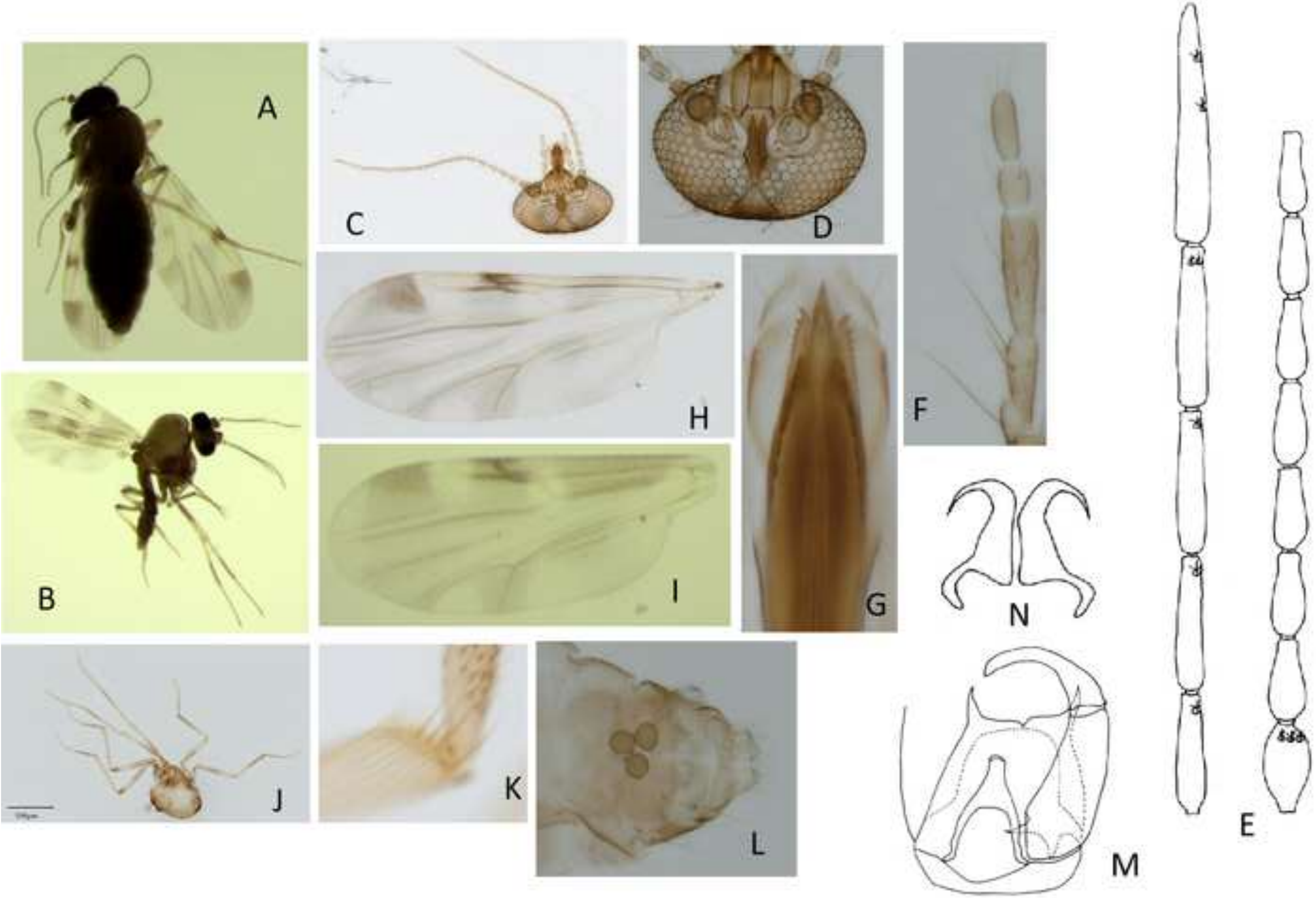

Figure 2

Click here to access/download;Figure;Figure 2.tif

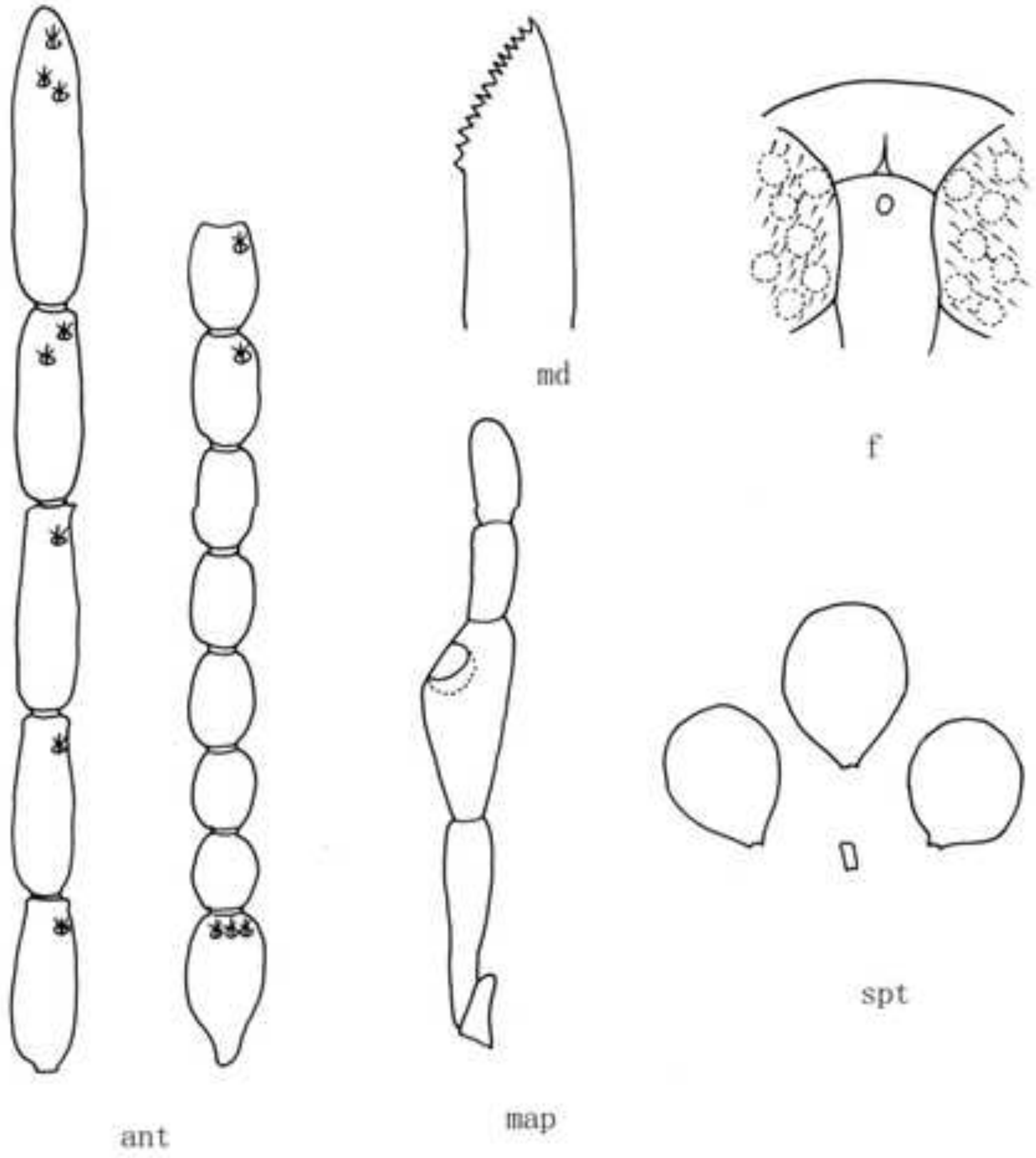

Figure 3

Click here to access/download;Figure;Figure 3.tif

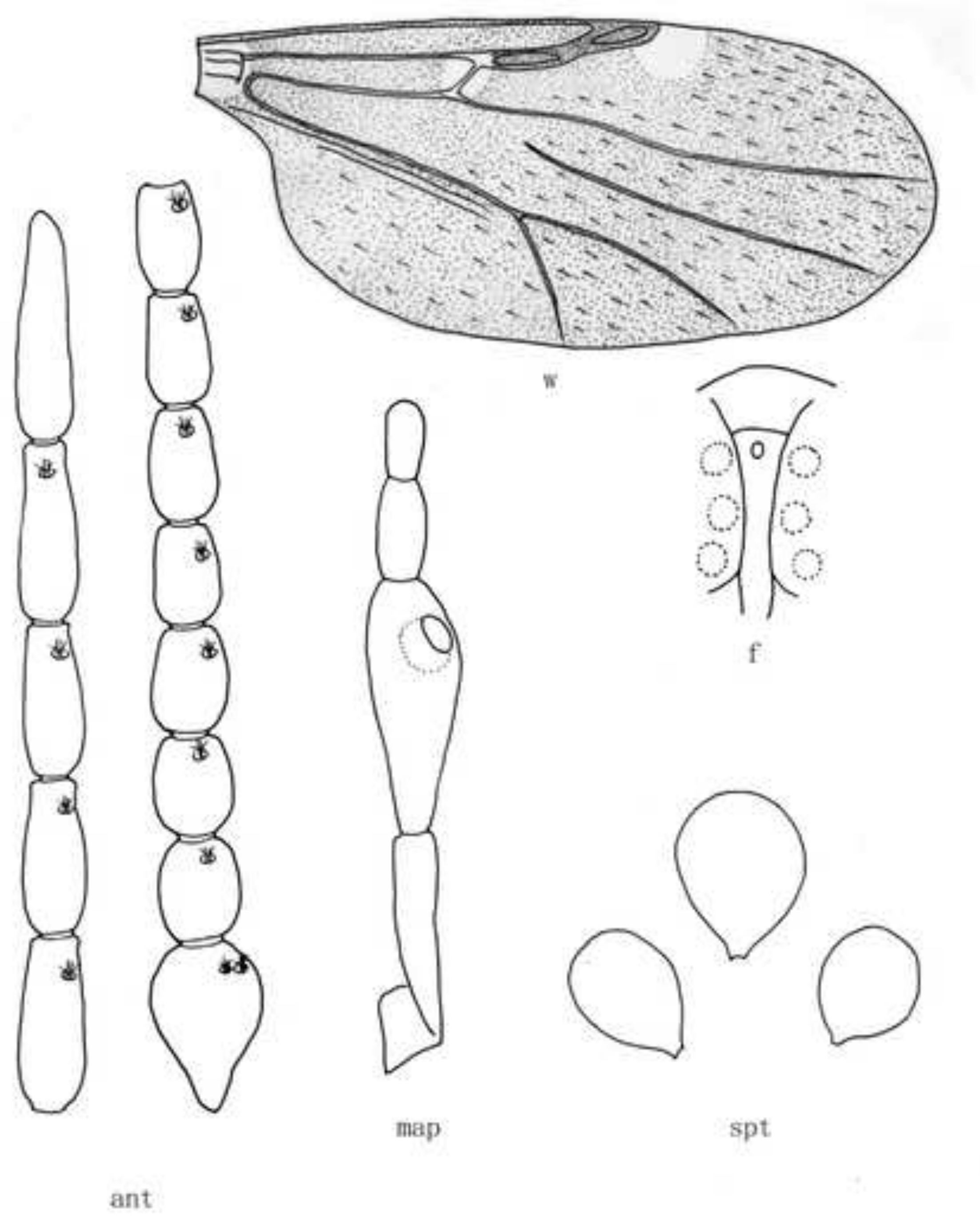

Figure 4

Click here to access/download;Figure;Figure 4.tif

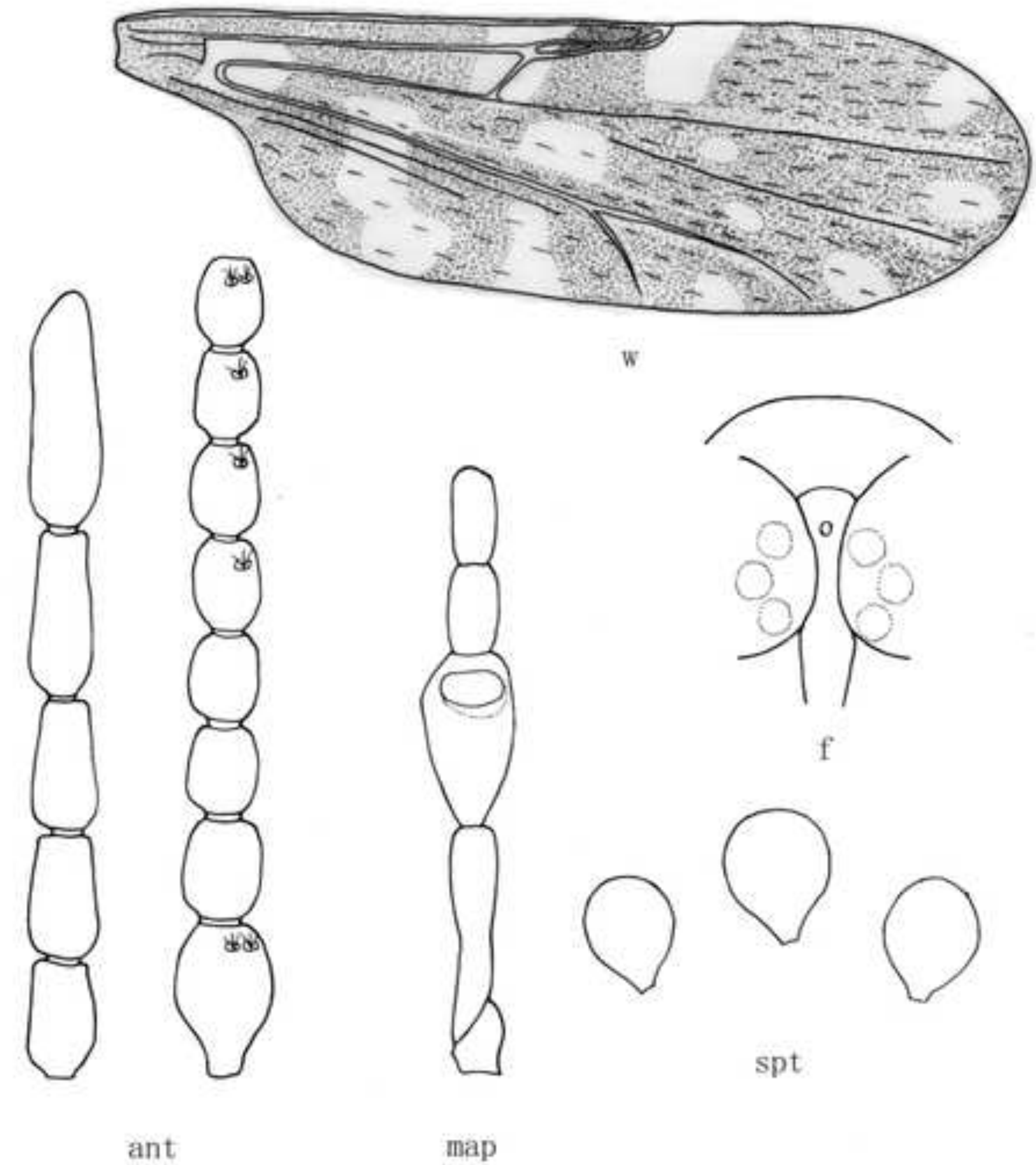

Figure 5

Click here to access/download;Figure;Figure 5.tif

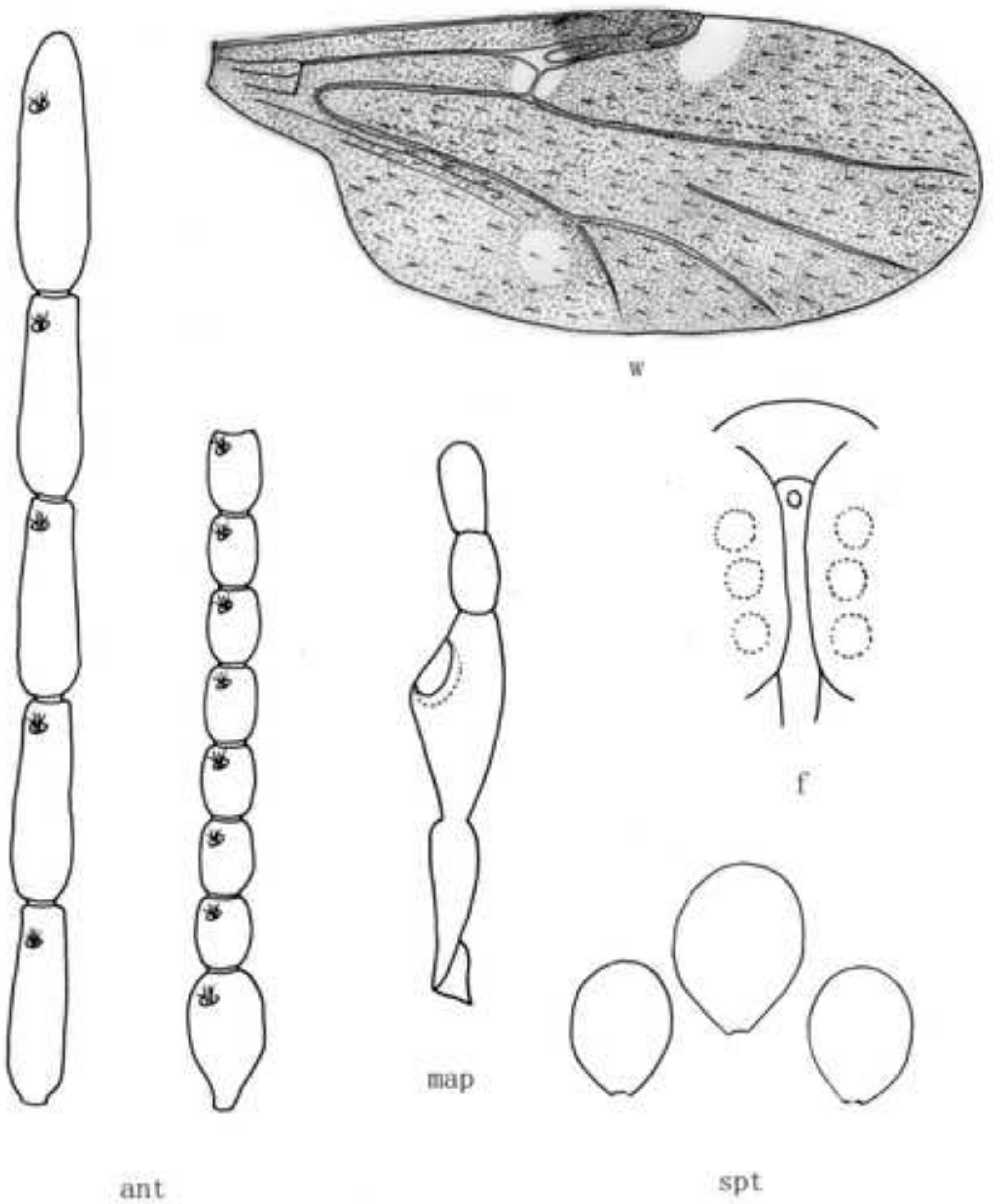

Figure 6

Click here to access/download;Figure;Figure 6.tif

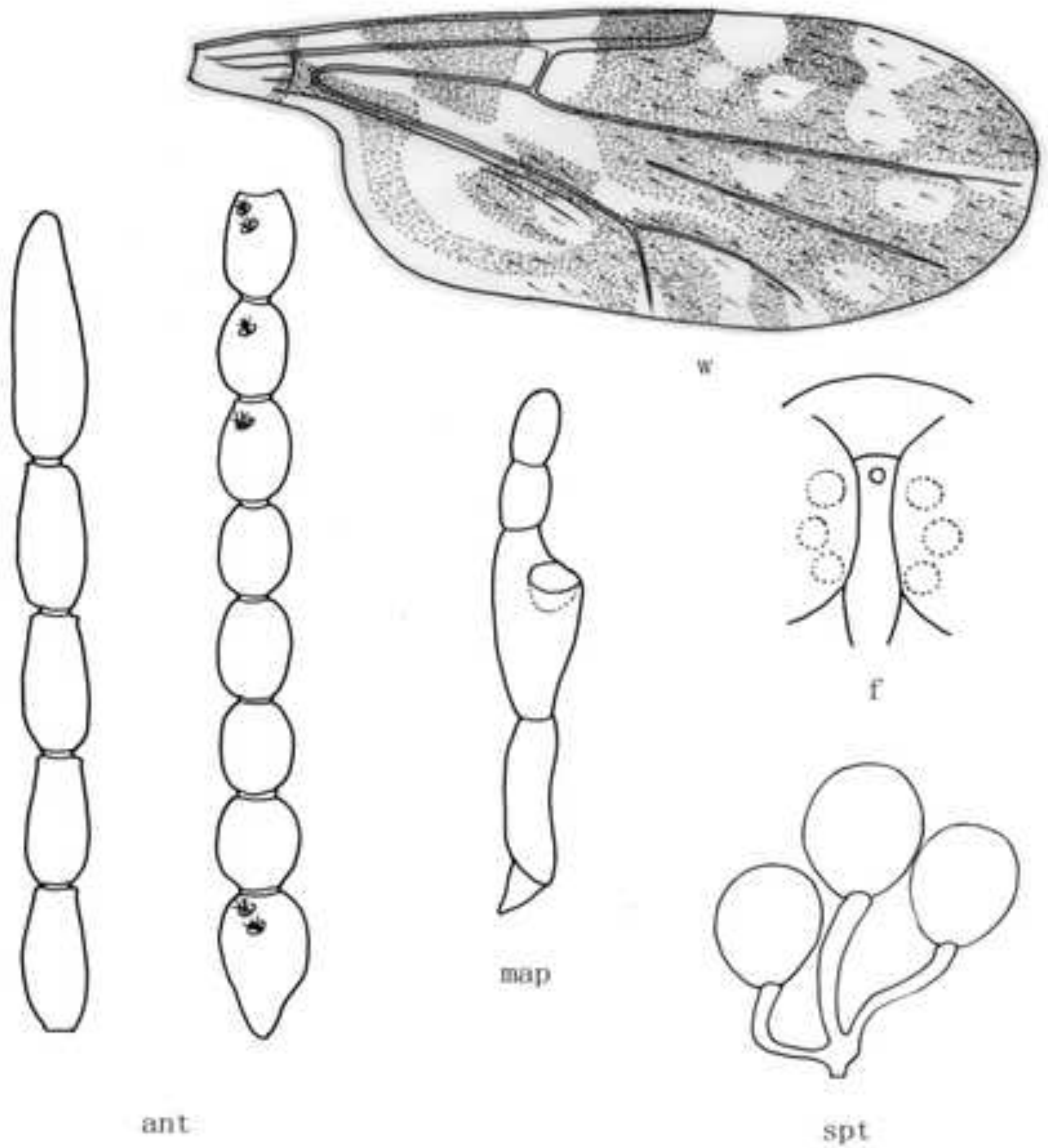

 Click here to access/download;Figure;Figure 7.tif

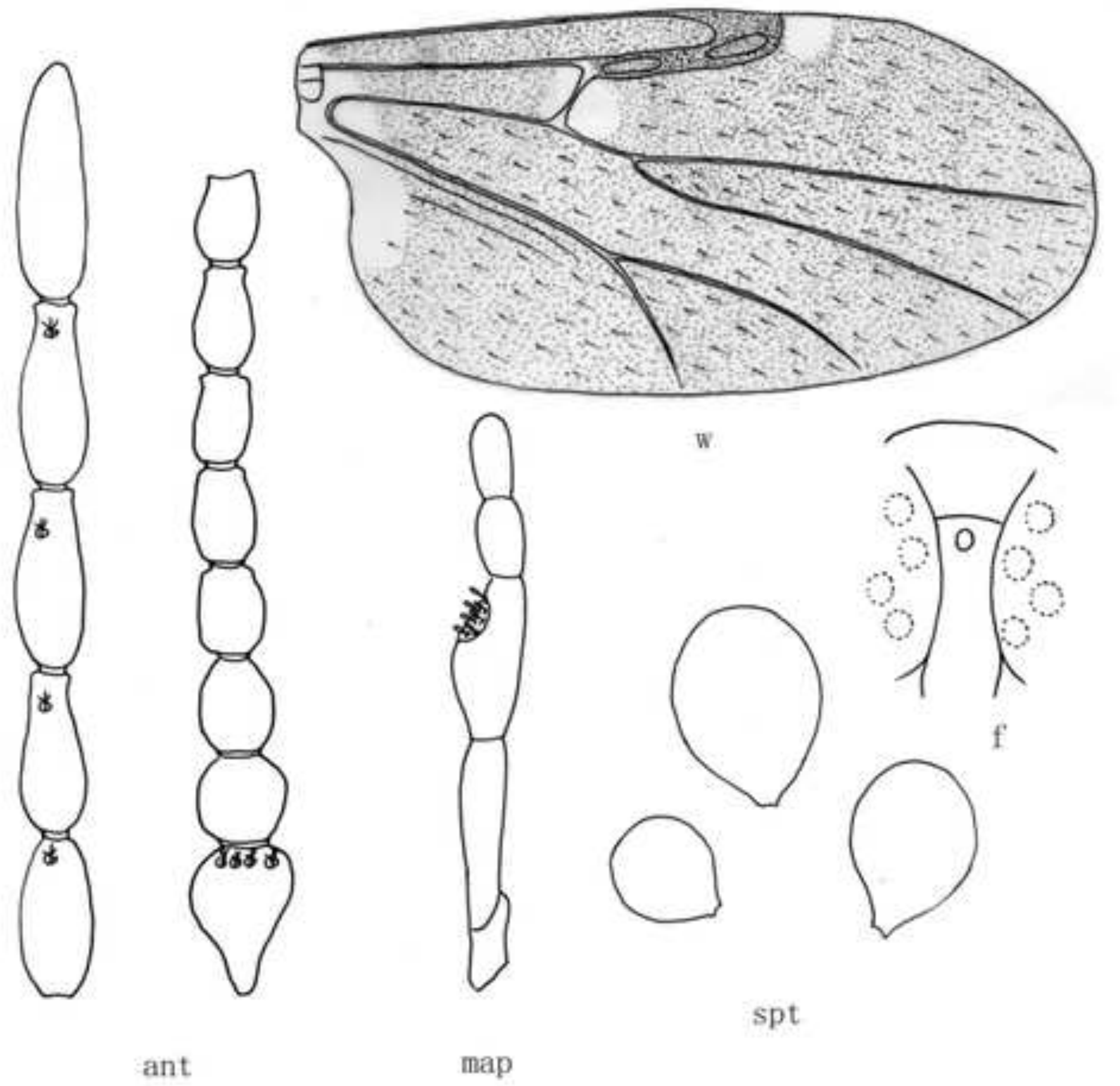

 Click here to access/download;Figure;Figure 8.tif

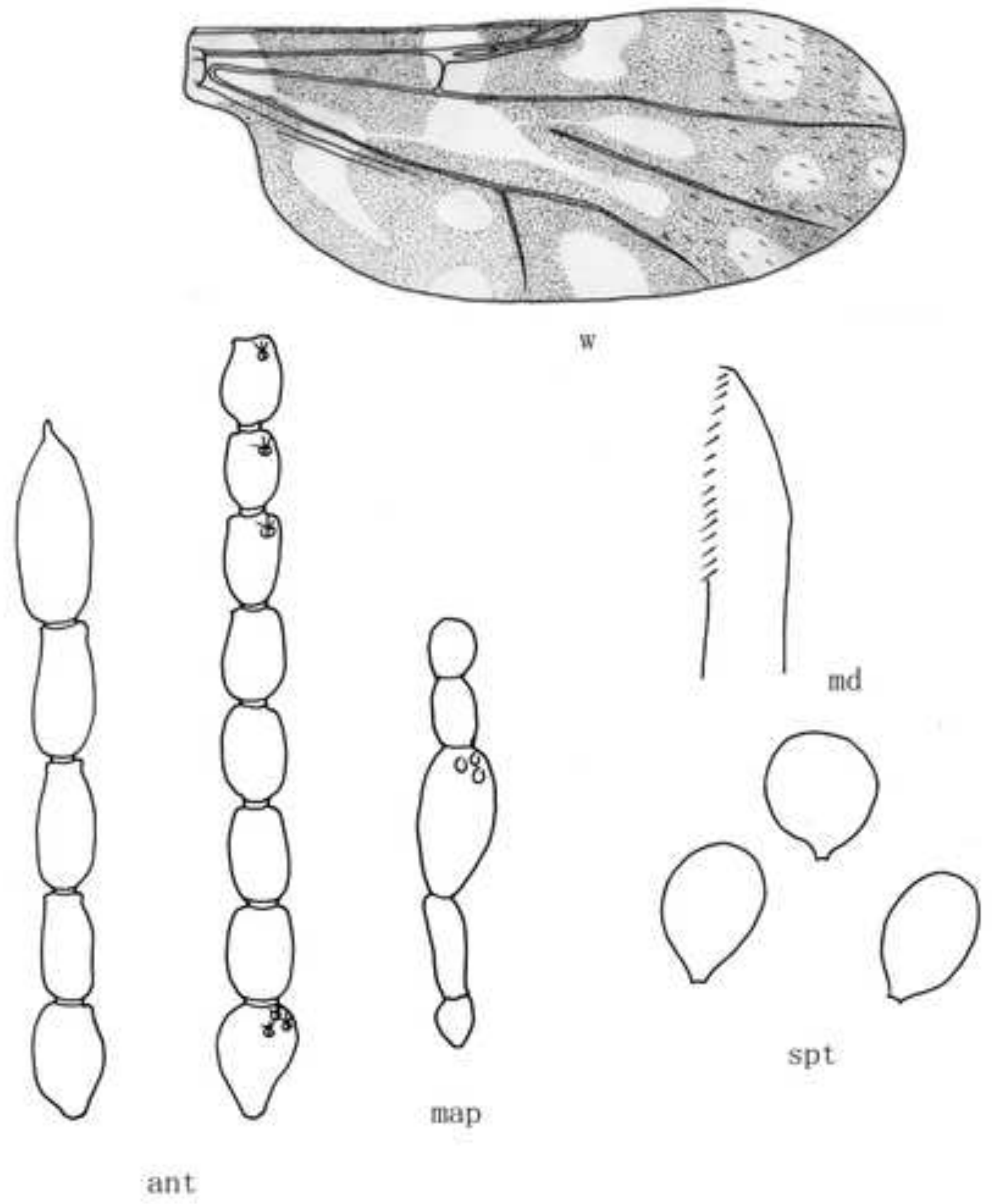

Figure 9

Click here to access/download;Figure;Figure 9.tif

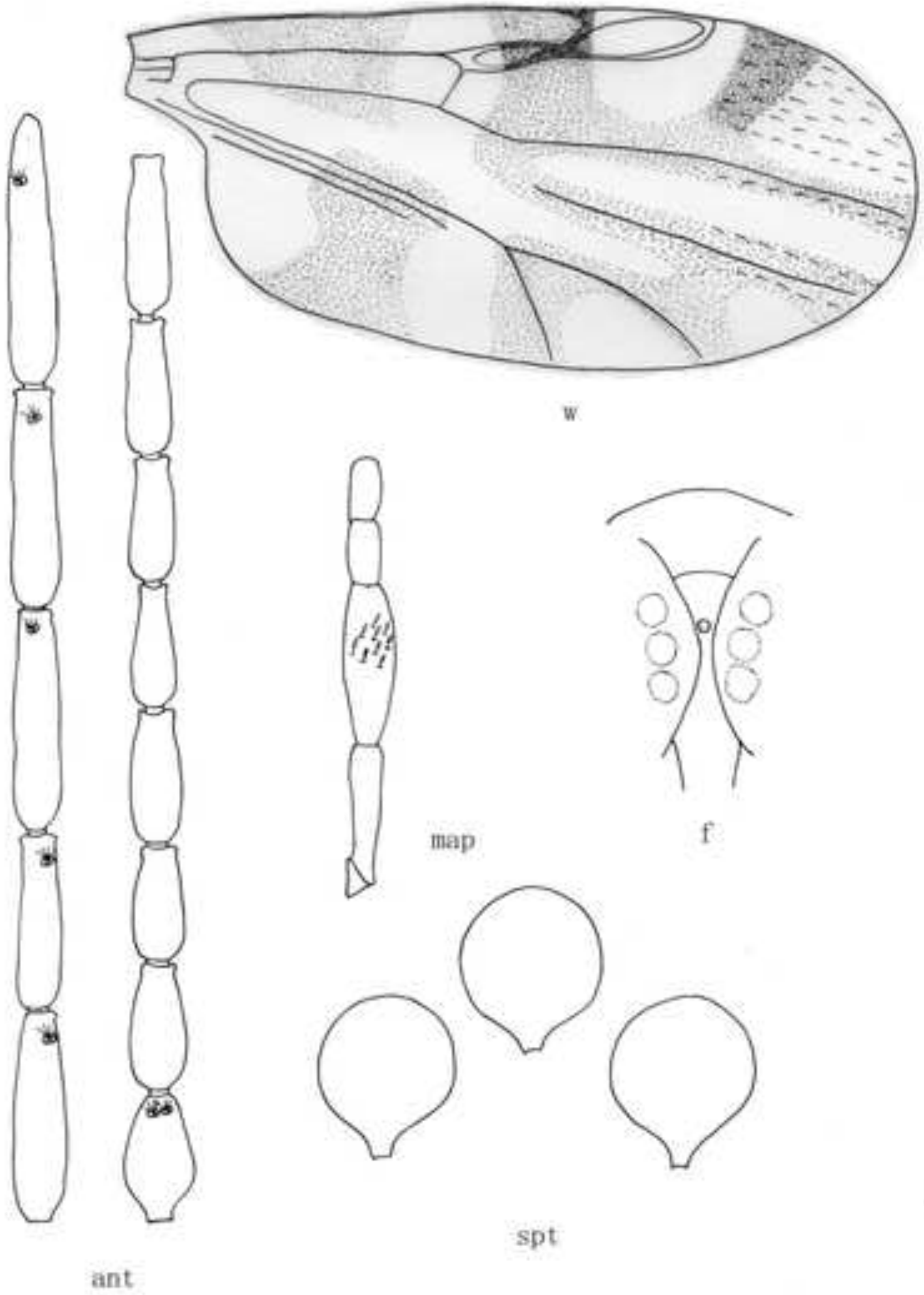

Figure 10

Click here to access/download;Figure;Figure 10.tif

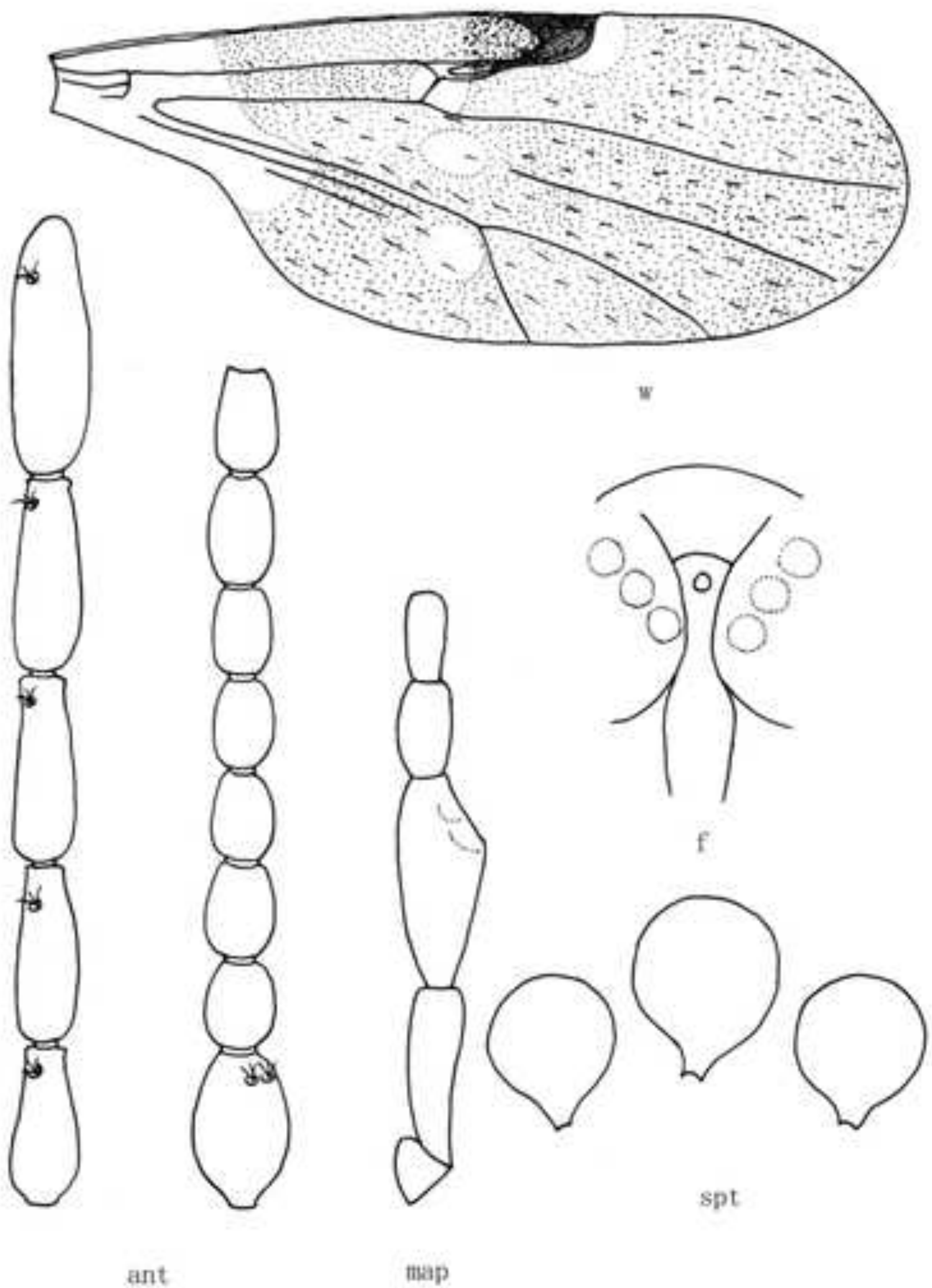

Figure 11 Click here to access/download;Figure;Figure 11.tif

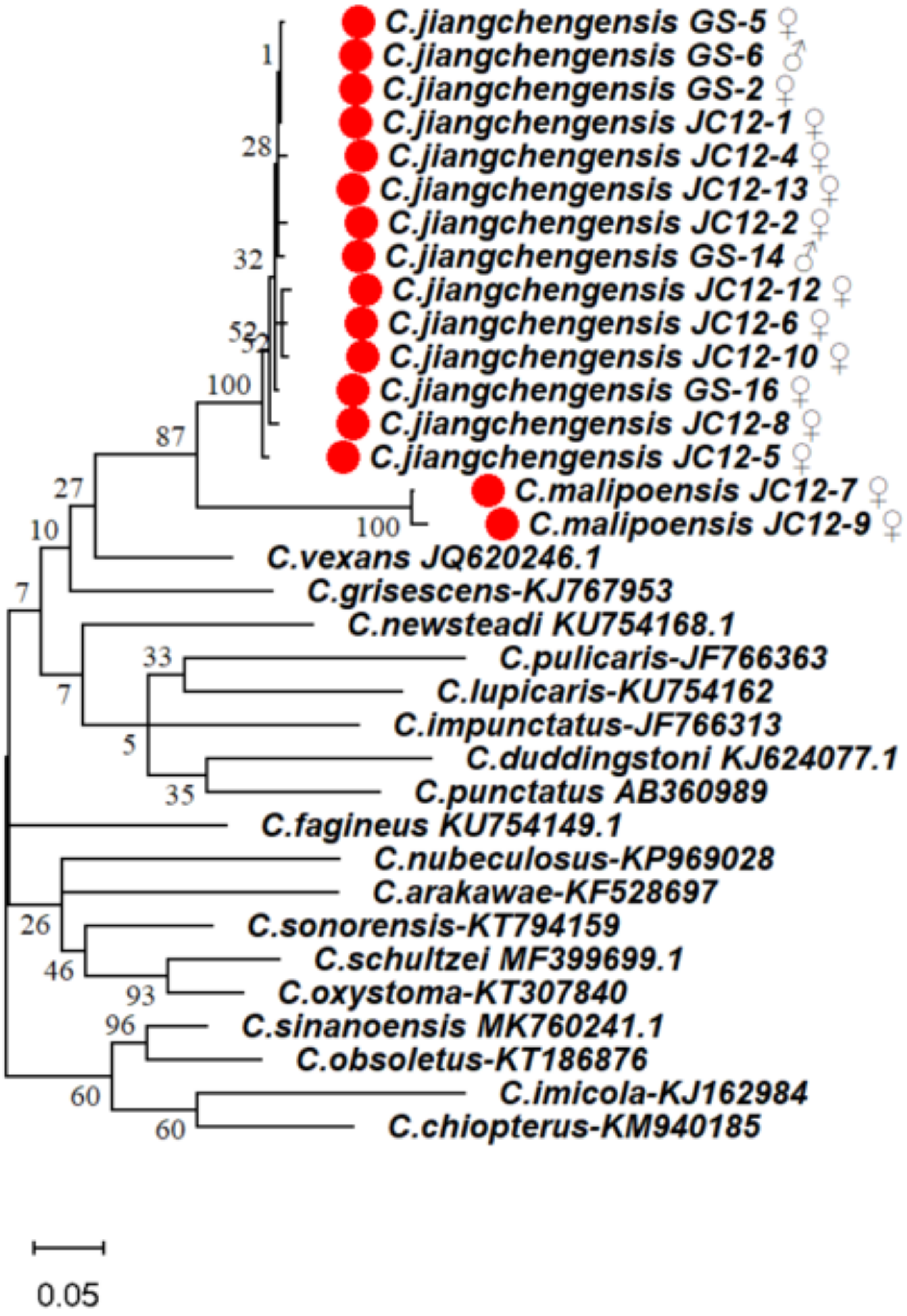

Supplement: Attachment — Submitted filename: PONE-D-22-32078_reviewer.pdf [file pone.0287266.s002.pdf]
