# Supplementary material for: Culicoides jiangchengensis, a new species of the subgenus Sinocoides (Diptera, Ceratopogonidae) based on integrative taxonomy from China
Source: PLoS One. 2023 Jul 26;18(7):e0287266. doi: 10.1371/journal.pone.0287266 (PMC10370730; doi:10.1371/journal.pone.0287266)
Supplement: Attachment — Submitted filename: response to reviewer_20230218.docx [file pone.0287266.s003.docx]

Dear Bi-Song Yue, Ph.D

Thank you for your letter (on 23th December 2022) and for the referees’ thoughtful comments concerning our manuscript entitled " Culicoides jiangchengensis, a new species of the subgenus Sinocoides (Diptera, Ceratopogonidae) based on integrative taxonomy from China” (PONE-D-22-32078).

We have studied reviewer comments carefully and have also addressed the comments as described below and made corrections on the revised version that we hope meet with their approval.

Thank you very much for your consideration.

Sincerely yours,

Jinglin Wang, Ph.D.

Yunnan Tropical and Subtropical Animal Viral Disease Laboratory,

Yunnan Animal Science and Veterinary Institute, Kunming, Yunnan province.

Qinglongshan, Jindian, Pang Long District, Kunming 650224, P.R. China

Tel: 86-871-6501-5606

Fax: 86-871-6501-0043

E-mail: wangjl107@163.com

**Response**

1.When submitting your revision, we need you to address these additional requirements.

**Answer:** I have carefully modified the format of the article according to the PLOS ONE's style requirements, carefully checked grant numbers, deleted the Funding Statement in the Acknowledgments Section, moved the ethics statement to the Methods section, and properly registered a new zoological taxon of the Culicoides.

**Funding Statement:** This work is supported by grants from the Basic Research Projects of Yunnan Province (grant no. 2019FA015 and 202201AS070062), National Natural Science Foundation of China (grant no. 32260896 and 81960605), Scientific and technological innovation team construction project of Kunming Medical University (grant no. CXTD202111), Projects funded by the central government to guide local scientific and Technological Development (202207AB110006), and Yunnan Chenggong expert workstation (202005AF150034). The funders had no role in the study design, data collection and analysis, decision to publish, or preparation of the manuscript.

**Reviewer #1:**

1.- Abstract, I suggested to modify the first sentence as “Biting midges of the genus Culicoides are medical and veterinary important because ….”

**Answer:** According to the comment, we have modify the first sentence in the new manuscript。
2.- Abstract, 3rd line, change “The” to “the”

- Abstract, 4th line, remove “both sexes”
- Abstract, 5th line, remove “in detail”
- Abstract, second page, 2nd and 3rd lines, Diptera, Ceratopogonidae should not italic, and remove underline from Culicoides.

**Answer:** According to the comment, these errors in the abstract have been modified in the new manuscript.

3.- Introduction, 1st line, add “causing agents” after disease, vectors do not carry disease, they carrying disease causing agents.

**Answer:** According to the comment, we have revised this sentence in the new manuscript.

3.- Materials and methods, more details of PCR should be provided e.g. primers, reaction conditions. Also, details of PCR product purification and sequencing must be provided. In addition, the details of MEGA-X software analysis must be provided e.g. analysis what? criteria setting for data analysis?

**Answer:** According to the comment, we have added the above contents in the new manuscript.

5.- Diagnosis, I am wondering why “only for Culicoides species in Yunnan”? Why not for Culicoides in other regions? Please explain this.

**Answer:** we have deleted the word “in Yunnan” in the new manuscript.

6.- Discussion, it will be very useful to add more details of morphological characteristics that a new species is different from previously described species. For examples, “C. jiangchegensis

1 ) without sensory pit in 3rdsegment of palpus;

2 ) sensilla coeloconica on flagellomere 1, 9-13;

3 ) m1 and m2 cell of the wings with pale spots, which were different from the other eight species of culicoides in subgenus Sinocoides, such as C. anthropophygas (with sensory pit in 3rd segment of palpus???, sensilla coeloconica on flagellomere ?????, m1 and m2 cell of the wings with ???? spots,)…” Details of morphological comparison like this should also provide for other species, particularly for morphologically closely related species, C. malipoensis.

**Answer:** More details of morphological characteristics that a new species is different from previously described species have been described in the section Remarks.

7.- Figure 11, phylogenetic tree, the outgroup species should be included in the phylogenetic analysis. Bootstrap values lower than 50% should not show on the figure.

**Answer:** The phylogenetic tree was constructed based on the COI gene sequences of C. jiangchengensis, C. malipoensis from Jiangcheng County and Gongshan County, Yunnan Province and another 24 species of Culicoides. These other 24 species of Culicoides can be used as the outgroups species. According to the comment, bootstrap values lower than 50% should not show on the figure 11.

8.- English should be check again by native speaker.

**Answer:** We have modified the English Language in the new manuscript. The English in this document has been checked by at least two professional editors, both native speakers of English. For a certificate, please see:

http://www.textcheck.com/certificate/WoMsXU

9. Reviewer #2: I think that this type of studies should be published more in taxonomy journals. In this study, the existence of a new Culicoides species, its place in classification and its morphological features were described. Therefore, I think this research paper is not suitable for PLOS ONE.

**Answer:** I agree with the Reviewer #2 that our study is about taxonomy. However, considering that PLOS ONE is a high-level comprehensive magazine, we think that our study suitable for PLOS ONE, so we submitted the manuscript into the journal.

10. Reviewer #3: The submitted manuscript provides very interesting and significant results. However, the text is not checked very carefully and it consists of many small technical mistakes. English should be revised. Structure of the manuscript should be modified in the direction to be simple to follow and to read. Descriptions should be given within the regular English structure of sentences. Verbs are missing in the description text.

**Answer:** We have read a lot of literature on the morphological characteristics of animals. When describing the morphological characteristics of animal species, verbs are missing in the description text so that readers can quickly capture the information of animal morphological characteristics. The English in this manuscript has been checked by at least two professional editors, both native speakers of English. For a certificate, please see:

http://www.textcheck.com/certificate/WoMsXU

Specific comments are given below:
11.Abstract
“blood-sucking feeding habits”: it is not the habit but the blood-feeding regime or haematophagous feeding regime.
**Answer:** According to the comment, we have revised it in the new manuscript.

12.The morphological: small letter
culicoides: capital letters. Culicoides should be in italic. If authors wanted to write in English than is biting midges
Genetically, C. jiangchengensis most closely related to C. malipoensis: missing verb … are most closely related to….
Diptera: Ceratopogonidae: this should not be in italic.
**Answer:** According to the comment, we have revised them in the new manuscript.

13.Introduction
that transmit arboviruses …. is enough
This cannot be cited this way “Liu et al. (1990, 1996, 2003)” because first is Liu and Yu and other two are Liu et al.
Same comment as previous for Liu et al. (2006, 2011) and Wu et al. (2018).
**Answer:** According to the comment, we have revised them in the new manuscript.

14.Material and methods
august big lletter
Diagnosis: this text should be significantly improved because in this way is very difficult to follow and to read.
**Answer:** According to the comment, we have revised them in the new manuscript.

15.Page 13: Please delete redundant space
C.jiangchengensis: here is missing space
Table 2. Abbreviations should be given below the table or in the title
Figure titles are missing spaces

**Answer:** According to the comment, we have revised them in the new manuscript.

16. Reviewer #4: This manuscript reports a new species of the biting midge subgenus Sinocoides of the genus Culicoides by using integrative approaches from morphological and molecular evidence. The authors provide detail description and illustrations of the morphological characters of the new species and other described species of the same subgenus. The identification appears to be accurate. In general, the manuscript is technically solid. I made some minor notes in the pdf file of the manuscript. Besides, I suggest the authors provide more details about the molecular phylogenetic analysis. For example, in Material and Methods, please provide how the sampling was designed and what the analytical methods they used for the phylogenetic analysis.

**Answer:** According to the comment, we provide more details about the molecular phylogenetic analysis in Material and Methods. some minor notes in the pdf file of the manuscript have revised in the new manuscript.
