# Supplementary material for: Culicoides jiangchengensis, a new species of the subgenus Sinocoides (Diptera, Ceratopogonidae) based on integrative taxonomy from China
Source: PLoS One. 2023 Jul 26;18(7):e0287266. doi: 10.1371/journal.pone.0287266 (PMC10370730; doi:10.1371/journal.pone.0287266)
Supplement: Attachment — Submitted filename: response to reviewer_20230502.docx [file pone.0287266.s004.docx]

Dear Rose Ann Joyce Sagun Puetes

Thank you for your letter (on may 2，2022) and for the referees’ thoughtful comments concerning our manuscript entitled " Culicoides jiangchengensis, a new species of the subgenus Sinocoides (Diptera, Ceratopogonidae) based on integrative taxonomy from China based on integrative taxonomy from China” (PONE-D-22-32078R2).

We have studied reviewer comments carefully and have also addressed the comments as described below and made corrections on the revised version that we hope meet with their approval.

Thank you very much for your consideration.

Sincerely yours,

Jinglin Wang, Ph.D.

Yunnan Tropical and Subtropical Animal Viral Disease Laboratory,

Yunnan Animal Science and Veterinary Institute, Kunming, Yunnan province.

Qinglongshan, Jindian, Pang Long District, Kunming 650224, P.R. China

Tel: 86-871-6501-5606

Fax: 86-871-6501-0043

E-mail: wangjl107@163.com

**Response**

1. We note that several of your files are duplicated on your submission. Please remove any unnecessary or old files from your revision, and make sure that only those relevant to the current version of the manuscript are included.

**Answer:** We have removed the unnecessary or old files in our new submissions.

2. We notice that your manuscript file with Track Changes was uploaded on February 21, 2023. Please can you upload the latest version of your highlighted version as Revised Manuscript with Track Changes.

**Answer:** we have uploaded the latest version of your highlighted version as Revised Manuscript with Track Changes.

3. Please ensure that you refer to Table 2 in your text as, if accepted, production will need this reference to link the reader to the Table.

**Answer:** According to the comment, We have refereed to Table 2 in the new manuscript.

4. Please amend your current ethics statement to confirm that your named institutional review board or ethics committee specifically approved this study.

**Answer:** According to the comment, We have amended the current ethics statement to confirm that our named institutional review board or ethics committee specifically approved this study.

**Response to reviewer on 17^th^ April 2023**

1.Again Diptera and Ceratopogonidae should not be in italic.

**Answer:** According to the comment, we have modify them in the new manuscript

2.Ceratopogonidae transmit viruses and not one virus. Please correct that in the first sentence of introduction.
**Answer:** According to the comment, we have modify the first sentence of introduction in the new manuscript.

3.Please correct “china” to capital letter
**Answer:** According to the comment, we have corrected “china” to capital letter in the new manuscript.

4.Dallas et al. (2003) should all go into brackets.
**Answer:**According to the comment, we have put “Dallas et al. ( 2003 )” all in brackets.

5.culicoides should be in capital letter

**Answer:** According to the comment, we have revised it in the new manuscript.
